# Tuning Epithelial Cell–Cell Adhesion and Collective Dynamics with Functional DNA-E-Cadherin Hybrid Linkers

**DOI:** 10.1021/acs.nanolett.1c03780

**Published:** 2021-12-23

**Authors:** Andreas Schoenit, Cristina Lo Giudice, Nina Hahnen, Dirk Ollech, Kevin Jahnke, Kerstin Göpfrich, Elisabetta Ada Cavalcanti-Adam

**Affiliations:** † Biophysical Engineering Group, Max Planck Institute for Medical Research, Jahnstraße 29, D-69120 Heidelberg, Germany; ‡ Department of Cellular Biophysics, Growth Factor Mechanobiology Group, Max Planck Institute for Medical Research, Jahnstraße 29, D-69120 Heidelberg, Germany; ⊥ Department of Physics and Astronomy, Heidelberg University, D-69120 Heidelberg, Germany

**Keywords:** cell−cell adhesion strength, E-cadherin, DNA nanotechnology, adherens junction, epithelial cells, collective migration, DNA−protein hybrid, mechanotransduction

## Abstract

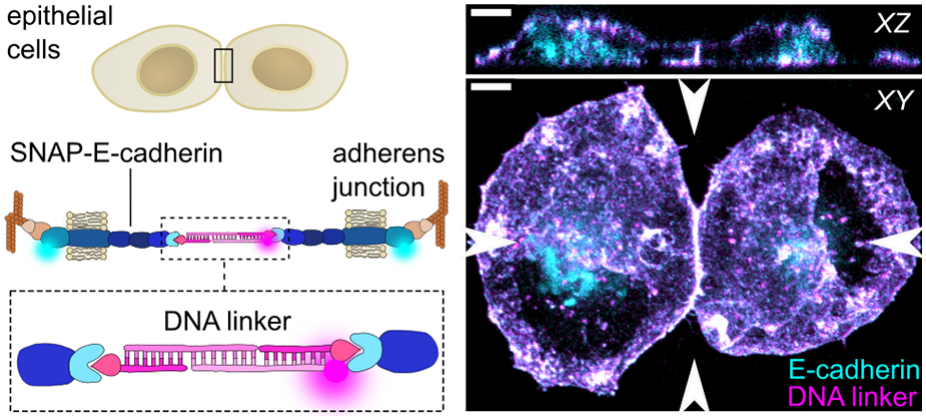

The binding strength between epithelial cells is crucial for tissue integrity, signal transduction and collective cell dynamics. However, there is no experimental approach to precisely modulate cell–cell adhesion strength at the cellular and molecular level. Here, we establish DNA nanotechnology as a tool to control cell–cell adhesion of epithelial cells. We designed a DNA-E-cadherin hybrid system consisting of complementary DNA strands covalently bound to a truncated E-cadherin with a modified extracellular domain. DNA sequence design allows to tune the DNA-E-cadherin hybrid molecular binding strength, while retaining its cytosolic interactions and downstream signaling capabilities. The DNA-E-cadherin hybrid facilitates strong and reversible cell–cell adhesion in E-cadherin deficient cells by forming mechanotransducive adherens junctions. We assess the direct influence of cell–cell adhesion strength on intracellular signaling and collective cell dynamics. This highlights the scope of DNA nanotechnology as a precision technology to study and engineer cell collectives.

Epithelial cells are linked to one another to maintain tissue structural integrity and to respond dynamically to events which require coordinated behavior, like morphogenesis or collective migration.^1^ Adherens junctions (AJs) mediate strong cell–cell adhesion and are especially important for the transduction of mechanical signals between cells, which govern collective dynamics.^2−5^ To establish the physical link, the adhesive receptors cadherins form *trans*-dimers with cadherins of neighboring cells. Epithelial cadherin (E-cadherin) is crucial for the epithelium integrity, and its loss is associated with different forms of cancer and the acquisition of invasive properties.^6^ E-cadherin dimerization leads to downstream signaling, which involves the recruitment of other AJ proteins, e.g., catenins, and remodeling of the actin cytoskeleton.^7,8^ At AJs, mechanical cues are translated into biochemical signals, which regulate fundamental cellular processes like proliferation and cell fate.^4,9^

The investigation of the influence of cell–cell adhesion and its mechanical regulation on these processes requires control over AJ assembly and functionality, which is mainly achieved by modulating AJ protein expression levels.^10^ Mutations in cadherins^11^ or RNA interference^12^ provide some control over cell–cell adhesion strength, but these approaches require extensive tuning depending on the cell type and experimental conditions. The depletion of calcium ions, required for cadherin dimerization,^13,14^ or recently reported optochemical and optogenetic approaches^15−17^ facilitate the spatiotemporal control over cell–cell adhesion assembly and disassembly. However, the influence of the molecular binding strength between cells remains unknown, since there is no method to precisely control it.

DNA nanotechnology allows for the programmable generation of molecular architectures with a sequence-tunable binding strength.^18,19^ Due to this versatility, combined with a large toolbox of chemical functionalization options, several applications have been presented in cell biology studies.^20^ DNA is commonly anchored on the cell membrane by hydrophobic moieties, like cholesterol or fatty acids that are covalently linked to the DNA.^21^ It has been used to facilitate artificial cell–cell adhesion in nonadherent or suspended cells,^22,23^ even with complex DNA nanostructures like DNA origami,^24^ which could be used to facilitate cell–cell communication.^25,26^ Furthermore, it has been reported that the control over the binding strength can be achieved by varying the DNA concentration.^27^ Moreover, DNA allows to program the cellular organization in bottom-up tissue assembly^28^ or to report forces within cellular monolayers.^29^ However, since DNA strands alone inserted into the cellular membrane do not interact with intracellular structures,^23^ the functionality of the artificial DNA link remains questionable. It is unclear whether downstream signaling is maintained upon artificial DNA-mediated cell–cell adhesion and its use in cell collectives remains largely unexplored.

Here, we present a functional DNA-E-cadherin hybrid to tune adhesion strength in epithelial cell collectives. By linking DNA to a truncated E-cadherin construct, we ensure a link with the intracellular machinery, while benefiting from the DNA sequence-dependent adhesion strength. Using force spectroscopy, we verify an increased cell–cell adhesion strength upon DNA linker addition, which can be reversed by using DNA strand displacement reactions. Furthermore, we demonstrate the recruitment of AJ proteins, which eventually leads to mechanosensing. Finally, we apply the DNA-E-cadherin hybrid to investigate the influence of cell–cell adhesion strength on collective dynamics in migrating epithelial monolayers.

First, we set out to achieve a precisely controllable semisynthetic, yet functional cell–cell adhesion linker for epithelial cells. While DNA-mediated cell–cell adhesion can be easily facilitated by a cholesterol-tagged DNA linker in nonadherent cells (Figure S1), this approach is not suitable for functional studies that require downstream signaling, like for epithelial cell collectives. We thus designed a DNA-E-cadherin hybrid system. Our approach combines the tunability and versatility of DNA with the intracellular signaling capabilities of the adhesive receptor E-cadherin, which binds via the AJ proteins catenins to the actin cytoskeleton (Figure 1A). *Trans-* and *cis-*clustering with other E-cadherins is facilitated by the two outer extracellular domains EC1 and EC2.^30^ Additionally, adhesion-independent E-cadherin clustering can be promoted, to a limited extent, by the actin cytoskeleton.^31^ To achieve control over the extracellular interactions involved in cell–cell adhesion, we replaced these domains with a SNAP-tag inserted into the sequence between R154 and N376, thereby maintaining the sequence for correct extracellular expression and further protein modification. The SNAP-tag allows fast and highly specific binding of any molecule functionalized with its ligand benzylguanine.^32^ The expression of SNAP-E-cadherin in A431D cells, which lack endogenous expression of E-cadherin,^33^ was successful but did not facilitate cell–cell adhesion (Figure S2A). We designed a 45 base pair long DNA linker consisting of identical benzylguanine-tagged anchor strands, which covalently bind to the SNAP-tag and hybridize with two complementary linker strands. The DNA linkers were specifically designed to provide stable and strong hybridization while being as close as possible to the natural E-cadherin in total length. Replacing two extracellular domains (∼14.7 nm) with the DNA and a SNAP-tag (∼17.5 nm) leads to an increased length of ∼2.8 nm of the completely assembled *trans*-DNA-E-cadherin hybrid dimer compared to a full-length *trans*-E-cadherin dimer, which is ∼38.5 nm long.^30^ The sequence-complementary part exhibits a calculated molecular binding strength of 17.7 kcal/mol.^34^ At 37 °C, all DNA linkers are completely assembled (Figure S3). Furthermore, the DNA was functionalized with a fluorophore (e.g., Cy5) for visualization (Figure 1A).

**Figure 1:**
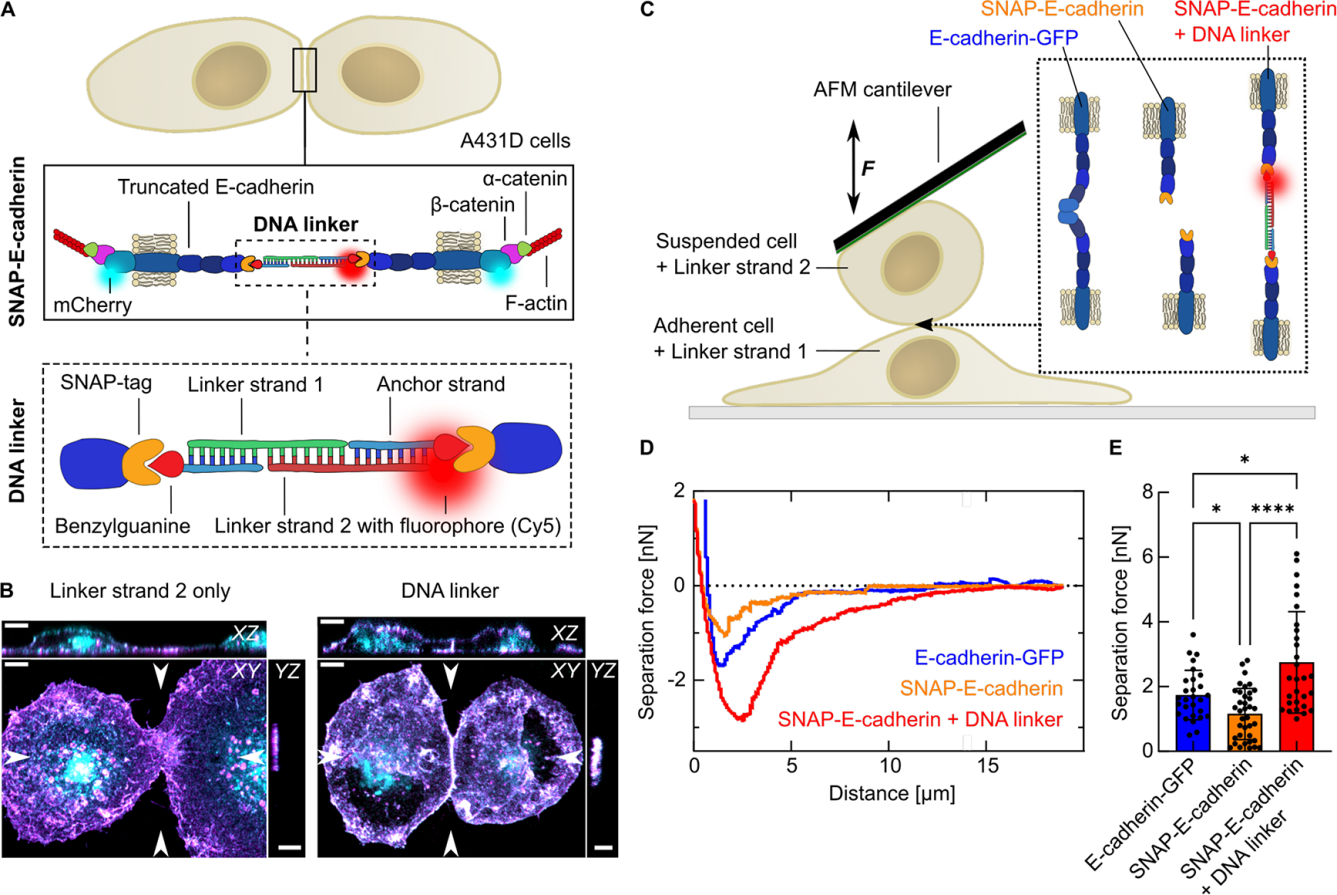
Cell–cell adhesion is facilitated by a DNA-E-cadherin hybrid linker. (A) Sketch of the DNA-E-cadherin hybrid linker system. Epithelial A431D cells express a truncated E-cadherin, where the extracellular domains EC1 and EC2 are replaced by a SNAP-tag. The intracellular domain is labeled with mCherry and binds via the proteins β-catenin and α-catenin to the F-actin cytoskeleton. SNAP-E-cadherins from neighboring cells form *trans-*dimers in the presence of the DNA linker. The SNAP-tag allows the binding of a 15 bp long anchoring DNA strand functionalized with benzylguanine. Two complementary 30 bp long linker strands are bound to the anchoring strand to form a duplex. The linker strands can be tagged with a fluorophore (e.g., Cy5) for visualization. (B) Whole-cell 3D reconstructions of A431D cells expressing SNAP-E-cadherin-mCherry (cyan) incubated with DNA linker strands (magenta). Cells were incubated with only linker strand 2 carrying Cy5 (left) or the complete Cy5-tagged DNA linker (right). Maximum projection and orthogonal slices through the positions indicated by arrows are shown for the mCherry and the Cy5 channel. Colocalization of E-cadherin and DNA linker results in white color. Scale bars, 5 μm. (C) Single cell force spectroscopy using AFM. Sketch of the experimental setup: Adherent cells are preincubated with Linker strand 1 for 1h. Suspended cells preincubated with Linker strand 2 are captured with the AFM cantilever. The cells are brought in contact and the separation forces are measured. (D) Representative separation force curves for cells expressing full-length E-cadherin-GFP (blue), SNAP-E-cadherin (orange) and SNAP-E-cadherin incubated with the DNA linker (red). The cell contact duration is 5 s. The separation force is the minimum of the curve. (E) Comparison of the separation forces. Bars show the mean value. Error bars show the standard deviation. Plots are generated from *N* = 3 independent experiments. Number of measured cells: *n*(E-cadherin-GFP) = 28. *n*(SNAP-E-cadherin) = 37. *n*(SNAP-E-cadherin + DNA linker) = 29. (*) *p*-value between 0.1 and 0.01. (****) *p*-value <0.0001. Multiple ANOVA tests with Welch’s correction. Alpha was set to 0.05.

The addition of only Linker strand 2 did not induce cell–cell contacts as no DNA duplex between two cells can be formed (Figure 1B, Figure S2A, Supporting Video S1). In contrast, the addition of the complete DNA linker led to an accumulation of both DNA (Figure 1B) and SNAP-E-cadherin construct (Figure S2A, Video 1) at the cell–cell interface. Furthermore, we observed the formation of a straight cell–cell junction with an increased height compared to the nonfunctional single linker strand. The DNA linker was stable at the cellular membrane for several hours, but its presence decreased over time, likely due to internalization of the DNA-E-cadherin hybrid receptors (Figure S2B,C). To demonstrate that DNA-mediated cell–cell adhesion on the molecular level translates to an increased cell–cell adhesion strength on the cellular level, we performed single-cell force spectroscopy measurements using an atomic force microscope (AFM). For this purpose, we probed the separation forces between a suspended cell bound to the AFM cantilever and an adherent cell^35,36^ (Figure 1C, Figure S4A). We compared A431D cells that either expressed full-length E-cadherin-GFP or the truncated SNAP-E-cadherin construct. In the latter case, adherent cells and suspended cells were separately preincubated with one of the complementary DNA linker strands, respectively. The captured cell was pushed on the adherent cell to probe cell–cell interactions, and the separation force was measured. The force spectroscopy measurements revealed that the truncated SNAP-E-cadherin, in the absence of the DNA linker, results in significantly weaker cell–cell adhesion than the E-cadherin-GFP (1.7 ± 0.8 nN versus 1.2 ± 0.8 nN, respectively). These values are in the same range as reported for other cadherin-dependent single cell force spectroscopy experiments in different cell types.^37,38^ Importantly, the addition of the DNA linker to SNAP-E-cadherin-expressing cells, and therefore the assembly of the DNA-E-cadherin hybrid system, led to a significantly increased adhesion strength of 2.8 ± 1.6 nN (Figure 1D,E). We observed this trend for different contact times (2, 5, and 10 s, Figure 1E, Figure S4B,C), which demonstrates that the DNA linker leads to fast and strong cell–cell adhesion.

Besides the controlled inducibility, another advantage of using a DNA linker is the reversibility of the linkage; i.e., the DNA duplex can be opened by toehold-mediated strand displacement.^39^ To enable strand displacement, we adapted the linker design by adding a 15 nucleotide long sequence overhang to Linker strand 2. This single-stranded DNA toehold-overhang cannot hybridize with Linker strand 1 since it is not complementary. Additionally, we designed an invader strand, which is complementary to Linker strand 2 including the toehold sequence. Its binding affinity to the toehold-modified Linker strand 2 is thus higher than the affinity between the two linker strands (Figure 2A). Added in excess (10×), the invader strand rapidly removed the toehold-modified linker strand within 1 min to open the DNA linker. The toehold-modified linker strand then diffuses out of the focal plane resulting in an increased background together with internalized strands (Figure 2B, Video 2). Hence, our approach provides temporal control to reversibly turn cell–cell adhesion on and off.

**Figure 2:**
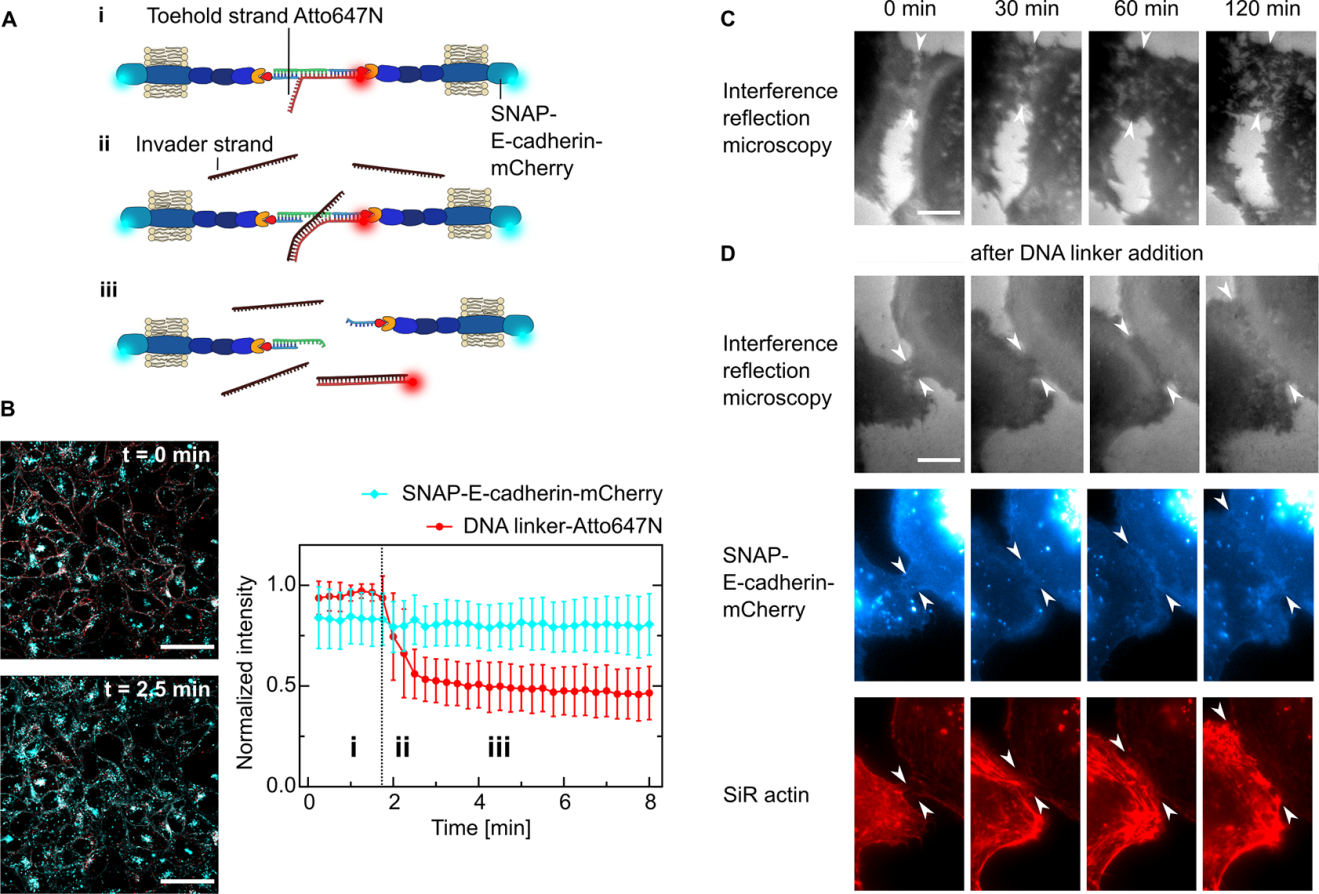
Dynamic control of cell–cell adhesion. (A) Sketch showing the reversibility of the cell–cell linkage by using toehold-mediated strand-displacement. A431D cells are linked with a DNA strand which contains a free overhang (toehold) functionalized with Atto647N (i). Upon addition of an invader strand in excess (ii), the linker strand is displaced and the link between the cells is broken (iii). (B) Representative confocal images of cells expressing SNAP-E-cadherin-mCherry (cyan) incubated with the DNA linker-Atto647N (red) before and after the addition of the invader strand. Scale bar, 50 μm. Quantification of the fluorescence intensity normalized to the maximum value over time of SNAP-E-cadherin-mCherry and DNA linker-Atto647N. The invader strand is added at *t* = 1.8 min, indicated by the dotted line. Mean values are plotted and error bars indicate the standard deviation. Plots generated from *N* = 3 independent experiments and *n* = 31 measurements. (C) Live-cell time-lapse snapshots of A431D cells expressing SNAP-E-cadherin-mCherry imaged by interference reflection microscopy without addition of the DNA linker. Images representative of *N* = 2 independent experiments. Scale bar, 10 μm. (D) Live-cell time-lapse snapshots showing the formation of a cell–cell junction between A431D cells expressing SNAP-E-cadherin-mCherry after the addition of the DNA linker: top row, interference reflection microscopy; middle row, SNAP E-cadherin (blue); bottom row, actin cytoskeleton labeled with SiR actin (red). Images are representative of *N* = 2 independent experiments. Scale bar, 10 μm.

Having demonstrated that the DNA-E-cadherin hybrid allows reversible cell–cell adhesion, we next determined whether the linkage between cells is functional and enables downstream signaling. Unlike commonly used strategies where cells were linked using sDNA functionalized with, e.g., hydrophobic tags but without a suitable transmembrane domain,^22−24,27,28^ we particularly designed the DNA-E-cadherin hybrid system to preserve the transmembrane and the intracellular cadherin domains which mediates the connection to the cytoskeleton. We therefore assessed the cellular reaction to the DNA linker addition qualitatively by live-cell microscopy. While the contact length between neighboring cells in the absence of the DNA linker did not change over time (Figure 2C), we observed a 3-fold increase of the contact length within the first hour after DNA linker addition. This was accompanied by an accumulation of SNAP-E-cadherin at the interface of the cells as well as actin remodeling, which points to an intracellular response to the extracellular signal (Figure 2D, Video 3).

Since the use of the DNA–protein hybrid enabled us to investigate, for the first time, the intracellular response to a DNA-based cell–cell linker, we visualized the subcellular localization of E-cadherin using stimulated-emission-depletion (STED) microscopy. Clustering of E-cadherin-GFP led to the formation of a pronounced and straight AJ (Figure S5A). No defined junction was detectable for cells expressing the nonfunctional SNAP-E-cadherin (Figure S5B). The addition of the DNA linker resulted in an accumulation of SNAP-E-cadherin at the cell–cell interface, resembling E-cadherin-GFP but with smaller and less organized clusters, reflecting the absence of the EC1 and EC2 protein domains and the contribution of the actin cytoskeleton (Figure S5C). Beyond receptor clustering, assembly of functional AJs is characterized by the presence of E-cadherin/β-catenin complexes, which establish a link via α-catenin to the actin cytoskeleton.^7^ Here, the E-cadherin/β-catenin complex is crucial for the mechanical stability of AJs^40,41^ (Figure 3A). Successful formation of AJs leads to actin remodeling, where the actin fibers are aligned parallel to the junction to stabilize it^7,42,43^ (Figure 3B). For the bare SNAP-E-cadherin, we could neither detect coclustering with β-catenin (Figure 3C) nor the parallel alignment of actin fibers at the cell–cell junction (Figure 3D). Thus, SNAP-E-cadherin by itself was not functional and did not facilitate AJ downstream signaling. In contrast, addition of the DNA linker triggered AJ-dependent signaling. Co-clustering with β-catenin (Figure 3E) as well as the actin distribution at the cell–cell junction (Figure 3F) were similar to the full-length E-cadherin, thus showing functional linkage between cells.

**Figure 3:**
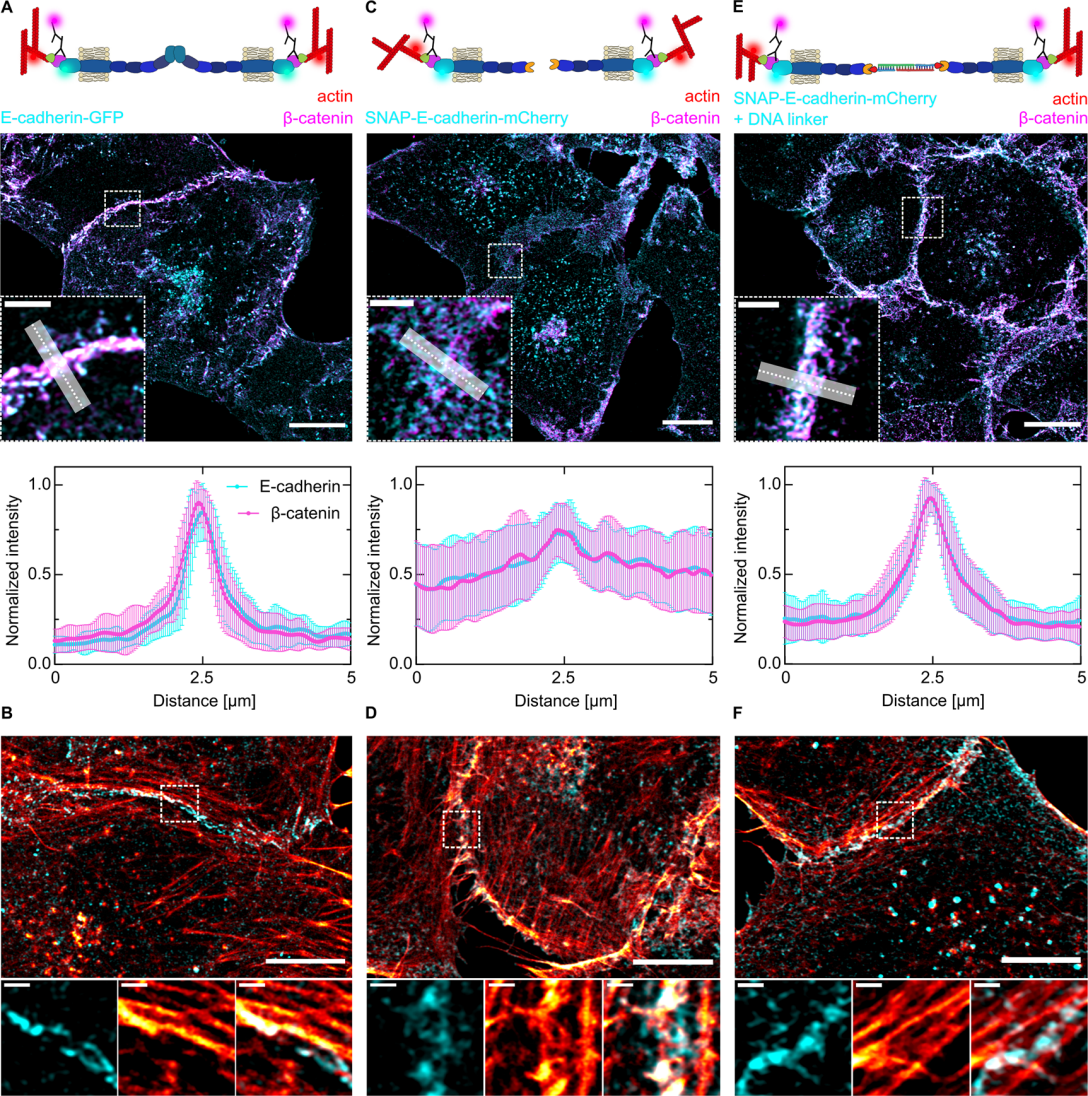
Downstream signaling of SNAP-E-cadherin after DNA linker addition. (A, C, E) Sketches of the different cell–cell adhesions between A431D cells expressing full-length E-cadherin-GFP (A) or SNAP-E-cadherin-mCherry without (C) or with the DNA linker (E). Airyscan confocal images of the subcellular localization of E-cadherin (cyan) and β-catenin (magenta) visualized by indirect immunostaining. Scale bars, 10 μm. Zoom-ins at the area indicated by the dashed line. Scale bars, 2 μm. Quantification of the fluorescence distribution normalized to the maximum intensity at the cell–cell interface generated by averaging the fluorescence intensity of multiple line plots (length = 5 μm, width = 1 μm). Error bars correspond to the standard deviation. *N* = 3 independent experiments and *n*(E-cadherin-GFP) = 20, *n*(SNAP-E-cadherin-mCherry) = 22, and *n*(SNAP-E-cadherin-mCherry + DNA linker) = 23 measurements. (B, D, F) Airyscan confocal images of the actin cytoskeleton (red) of A431D cells expressing full-length E-cadherin-GFP (B) or SNAP-E-cadherin-mCherry without (D) or with the DNA linker (F). Scale bars, 10 μm. Zoom-ins at the area indicated by the dashed line. Scale bars, 1 μm.

Mechanical signals are key for the determination of cell fate. An important function of AJs is sensing mechanical signals and transducing them from the outside to the inside of the cell via translation into biochemical signals.^44^ We thus investigated if our DNA-E-cadherin hybrid system is capable of mechanotransduction of intracellular downstream signaling. It is known that mechanotransduction involves the activity of the transcription regulator Yes-associated protein (YAP) and influences its subcellular localization.^4^ YAP is excluded from the nucleus and translocated to the cytosol upon phosphorylation, which is induced through mechanical cues from surrounding cells, sensed at functional and mechanically active AJs.^45,46^ We thus quantified YAP distribution for DNA-linked cells in comparison to the controls. In contrast to cells expressing E-cadherin-GFP, we observed the nuclear accumulation of YAP in cells expressing only SNAP-E-cadherin (Figure 4A). We assessed the nuclear to cytosolic ratio of YAP (Figure 4B) and quantified the fractions of areas within the monolayer where YAP was predominantly localized in the nucleus, the cytosol, or both (Figure 4C). The nuclear to cytosolic ratio increased from 0.7 ± 0.2 for E-cadherin-GFP expressing cells to 1.1 ± 0.3 when SNAP-E-cadherin was expressed. Since a low nuclear to cytosolic ratio indicates YAP exclusion, it shows that mechanotransduction is compromised in SNAP-E-cadherin expressing cells. The nuclear exclusion of YAP from cells in which the DNA-E-cadherin hybrid was assembled demonstrated that the mechanical signal from the cell peripheries was transduced into the cytosol and to the nucleus. Using DNA linkers with different hybridization strengths due to changes in their sequence and binding kinetics (3.2, 10.4, and 17.7 kcal/mol, Figure S3) revealed that in cells adhering with the 10.4 kcal/mol linker (ratio: 0.7 ± 0.1) mechanotransduction was more pronounced than in cells adhering with the 3.2 kcal/mol linker (ratio: 0.9 ± 0.1). This is reflected in the level of nuclear exclusion of YAP, which is similar to the one observed in cells expressing the full-length E-cadherin (Figure 4B,C). The strongest linker did not lead to a further decrease of the nuclear to cytosolic ratio (0.7 ± 0.1), indicating that cellular mechanotransduction was fully achieved with the 10.4 kcal/mol linker. Note that the only difference between the 3.2 kcal/mol and the 10.4 kcal/mol linker is an increased length of only two base pairs. Thus, the DNA-E-cadherin hybrid facilitates physiological outside-in signaling downstream of E-cadherin, which can be fine-tuned through subtle differences in the DNA hybridization strength.

**Figure 4:**
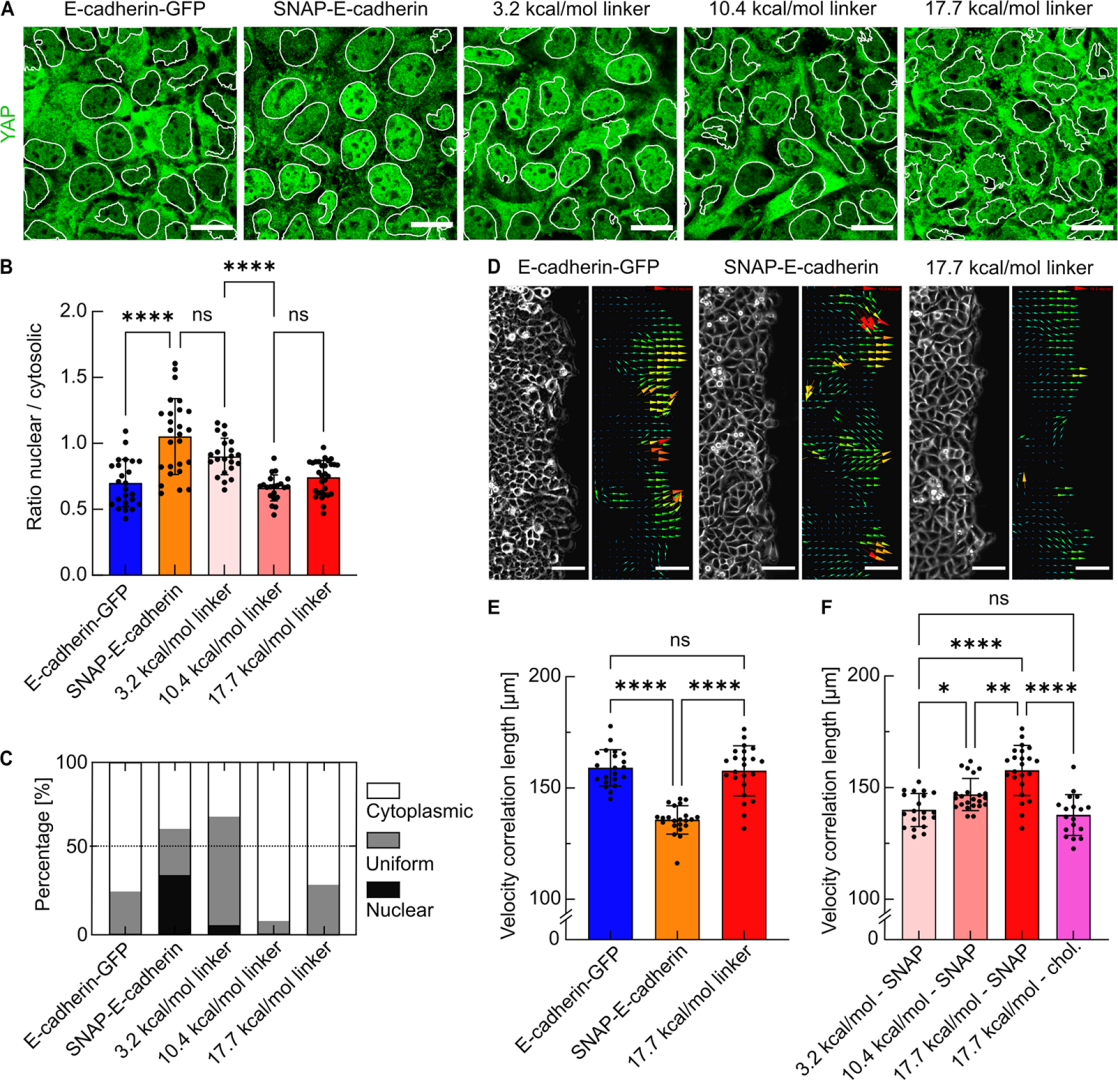
Effect of the DNA linker on cell–cell signaling and epithelial collective dynamics. (A) Confocal images showing YAP (green) with the nuclear segmentation (white outlines) for cells expressing E-cadherin-GFP or SNAP-E-cadherin after the incubation with DNA linkers of different hybridization strengths (3.2, 10.4, or 17.7 kcal/mol). Scale bars, 20 μm. (B) Quantification of the nuclear--to-cytosolic intensity ratios *r* of YAP. Each data point represents one field of view containing 25–30 cells. Bars show the means; error bars are standard deviations. (C) Quantification of the subcellular localization of YAP classified according to the ratios *r* between nuclear and cytosolic YAP intensities. Nuclear, *r* ≥ 1.15; uniform, *r* > 0.85; cytosolic, *r* ≤ 0.85. *N* ≥ 3, data derived from *n*(E-cadherin-GFP) = 24 measurements, *n*(SNAP-E-cadherin) = 25, *n*(SNAP-E-cadherin +3.2 kcal/mol linker) = 22, *n*(SNAP-E-cadherin +10.4 kcal/mol linker) = 21, *n*(SNAP-E-cadherin +17.7 kcal/mol linker) = 29. (D) Brightfield and vector fields generated by particle image velocimetry (PIV) of the migration front of A431D cells expressing E-cadherin-GFP, SNAP-E-cadherin, or SNAP-E-cadherin incubated with the 17.7 kcal/mol DNA linker. Scale bars, 100 μm. (E) Comparison of the velocity correlation lengths between migrating cells. Every data point shows the average correlation length of one field of view from *t* = 3. Five hours to *t* = 7.3 h after removing the confinement, corresponding to 24 time points. *n*_FOV_ shows the number of analyzed fields of view from at least three independent experiments. *n*_FOV_(E-cadherin-GFP) = 21, *n*_FOV_(SNAP-E-cadherin) = 22, *n*_FOV_(SNAP-E-cadherin + DNA linker) = 23. (F) Velocity correlation length for the variation of the DNA hybridization strength (3.2, 10.4, or 17.7 kcal/mol) as well as the anchoring method (SNAP-E-cadherin or direct functionalization of the anchor strand with cholesterol). *N* ≥ 3, *n*_FOV_(3.2 kcal/mol - SNAP) = 19, *n*_FOV_(10.4 kcal/mol - SNAP) = 23, *n*_FOV_(17.7 kcal/mol - SNAP) = 23 (same data set as in (E) shown again for better comparability), *n*_FOV_(17.7 kcal/mol - chol) = 18. ns = no significance. (*) *p*-value between 0.1 and 0.01. (**) *p*-value between 0.01 and 0.001. (****) *p*-value < 0.0001. Multiple ANOVA tests with Welch’s correction. Alpha was set to 0.05.

Since our approach allows us to modulate not only cell–cell adhesion but also associated downstream signaling in cells, we next investigated how this modulated strength would impact epithelial collective dynamics on the multicellular scale. Therefore, we performed collective migration assays to assess long-range interactions between cells, which are also crucial in, e.g., wound healing or morphogenesis.^2,3^ In cell monolayers expressing E-cadherin-GFP, we observed aligned trajectories of neighboring cells. Cells expressing only the SNAP-E-cadherin displayed more misaligned trajectories. Strikingly, the addition of the DNA linker restored the coordinated migration, demonstrating that the linkage by the DNA-E-cadherin hybrid system was translated to cell collectives (Figure S6A, Video 4). We quantified the collective dynamics by calculating the velocity correlation length for the different conditions (Figure S6B,C). It is a measure of coordinated motion inferred from vector fields mapped by particle image velocimetry^47^ (Figure 4D). The migration was analyzed for 7 h, because the DNA linker was mainly internalized at this point (Figure S6E). The expression of SNAP-E-cadherin resulted in a decreased correlation length of 135.8 ± 6.2 μm compared to cells expressing E-cadherin-GFP (159.4 ± 7.9 μm), in line with recent reports.^12,48^ The correlation length could be recovered completely through addition of the DNA linker (157.7 ± 10.9 μm, Figure 4E). The increased coordination between migrating cells demonstrated a large-scale transduction of mechanical forces^2,49^ facilitated by the DNA-E-cadherin hybrid system. To exploit the potential of DNA nanotechnology, we tested DNA linkers of different binding strengths (3.2, 10.4, and 17.7 kcal/mol). This revealed that the coordination between migrating cells directly depends on the molecular binding strength, since the correlation length increased from 138.3 ± 7.3 to up to 157.7 ± 10.9 μm when using stronger linkers (Figure 4F). Furthermore, we functionalized the cellular membranes with the 17.7 kcal/mol linker using cholesterol-tags, which did not result in an increased correlation length (137.7 ± 8.9 μm, Figure 4F). This proves that the E-cadherin component of the hybrid system is crucial for its functionality.

In conclusion, we present a novel approach to investigate cell–cell adhesion by combining the advantages of DNA nanotechnology and protein engineering. The molecular binding strength of the DNA-E-cadherin hybrid is freely tunable by using different DNA sequences but retains downstream signaling capabilities through the remaining domains of E-cadherin. Using single-cell force spectroscopy, we have demonstrated that the addition of the DNA linker leads to an increased cell–cell adhesion strength compared to the truncated E-cadherin. Furthermore, the simple addition of benzylguanine-tagged DNA to the culture medium provides reversibility and temporal control over cell–cell adhesion, since the linker strands can be removed again from the DNA-E-cadherin hybrid within seconds using strand displacement. Cell adhesion mediated by the DNA linker is fast and occurs within 1 h. We show that the DNA linker system provides E-cadherin downstream signaling in terms of the formation of E-cadherin/β-catenin complexes, actin remodeling, and mechanotransduction. Besides investigating cellular reactions, we demonstrate that our approach is suitable for studies on cell collectives, like monolayer migration.

Thus, the work presented here could be useful to directly assess the influence of cell–cell adhesion strength on tissue homeostasis^50^ and adhesion-dependent signaling like the Hippo-YAP/TAZ pathway,^4,44^ if endogenous cadherins are replaced by truncated cadherins fused to a SNAP-tag. The DNA linker length determines the intermembrane distance. Thus, the DNA-E-cadherin hybrid could be used to study the effect of this distance on downstream signaling. Moreover, the general concept of using a DNA–protein hybrid to tune molecular binding strengths could potentially be applied to investigate other binding-strength-dependent cellular processes, e.g., cell-matrix adhesion through the modification of integrin.^51^ Besides the molecular binding strength, other factors could influence downstream signaling processes, in particular clustering and spacing of receptors, as demonstrated for integrins.^52^ Since any DNA strand can be bound to SNAP-E-cadherin, our approach opens up the possibility to use DNA origami^24^ to link epithelial cells in a functional way. This would allow us to investigate the influence of controlled E-cadherin spacing^53,54^ or patterning^55^ and therefore *cis-*clustering on adhesion and signaling processes. Segregation of different receptor subtypes can impact their activation.^56^ It must be noted that the recruitment of other cadherins to the DNA-mediated adhesion site might be possible. Moreover, the mechanics of the environment, like substrate stiffness^57^ or different forces within a tissue or organism influence cell fate.^58^ Since we can control the transduction of mechanical signals within a monolayer, our approach might help to further elucidate the relationship between mechanosensing and cell fate decisions. In summary, our approach based on the DNA-E-cadherin hybrid system allows us to investigate (i) the immediate effect of a freely tunable molecular binding strength with temporal control and reversibility on different biological processes ranging from single cells to cell collectives, while (ii) maintaining the outside-in biochemical signaling activity of transmembrane receptors.

## Supporting Information Available

Materials and methods section, Figure S1–6 of cells linked by cholesterol-anchored DNA on their surface, expression of SNAP-E-cadherin-mCherry and localization of the DNA linker, melting curves of the DNA linkers, single cell force spectroscopy, super resolution images of E-cadherin at cell–cell contact, effect of the DNA-E-cadherin hybrid system on collective migration, and supporting video descriptions (PDF)
3D projections of single cells (AVI)
Toehold-mediated strand displacement (AVI)
Adherens junction formation (AVI)
Migration front of cell collectives (AVI)

## Supplementary Material

nl1c03780_si_001.pdf

nl1c03780_si_002.avi

nl1c03780_si_003.avi

nl1c03780_si_004.avi

nl1c03780_si_005.avi
